# Editorial: Smart nano-architectures for neuroscience and neuroengineering: from properties to applications

**DOI:** 10.3389/fnins.2024.1462078

**Published:** 2024-07-31

**Authors:** Nurul Akmal Che Lah, Nava Shmoel, Sonia Trigueros

**Affiliations:** ^1^Centre for Advanced Intelligent Materials, Universiti Malaysia Pahang Al-Sultan Abdullah, Gambang, Pahang, Malaysia; ^2^Faculty of Manufacturing and Mechatronics Engineering Technology, Universiti Malaysia Pahang Al-Sultan Abdullah, Pekan, Pahang, Malaysia; ^3^McGovern Institute for Brain Research, MIT, Cambridge, MA, United States; ^4^Department of Genetics Microbiology and Statistics, University of Barcelona, Barcelona, Spain

**Keywords:** nanoarchitectures, nanotechnology, neuroscience, diagnosis, theranostic

Nanotechnology, with its innovations in nanoarchitecture, has emerged as a powerful tool for addressing major global challenges. From revolutionizing healthcare to bolstering food security, its potential is vast. Despite these advancements, numerous unresolved issues remain, particularly concerning their potential role in supporting cancer development and progression. The scientific pursuit is complex, with researchers investigating whether a unified theory could link continuum/molecular-level quantum mechanics with neural circuitry theory. Such a theory would provide a crucial framework for further safe and effective enhancements in performance.

This Research Topic represents collective efforts in neuroscience and recent advanced smart nanoarchitecture technologies, addressing various key concerns in both fields. A recent advancement is the use of nanoarchitecture for the precise diagnosis of Glioblastoma multiforme (GBM), the most aggressive type of brain glioma, as presented by Wei et al. Studies comparing organic nanomaterials known for their high biocompatibility and biodegradability, such as liposomes, polymer nanoparticles, and extracellular vesicles, have demonstrated promising prospects for clinical use. This review acknowledges the challenges in developing effective functional organic nanomaterials for GBM treatment and emphasizes the need to scale up production and address ethical issues related to nanotechnology.

In a case-control study, Macintosh et al. explored the effects of leukodystrophy-associated Pol III subunits on the migration, proliferation, differentiation, and myelination processes during oligodendrocyte maturation. The study revealed that reduced Pol III activity hindered the differentiation of these precursor cells into mature oligodendrocytes. The findings provide insight into the role of Pol III in oligodendrocyte development and shed light on the pathophysiological mechanisms underlying hypomyelination in POLR3-related leukodystrophy.

Maeng et al. explored the use of adrenal magnetothermal stimulation via magnetic nanoparticle (MNP) technology to study changes in the stress-induced hypothalamic-pituitary-adrenal axis and sympathoadrenal-medullary system. This approach demonstrated MNP-triggered adrenal release, evidenced by changes in physiological (heart rate) and serum markers (epinephrine and corticosterone) before and after conditioning. Beyond their application for probing peripheral organ function, magnetic nanomaterials have been increasingly recognized for their therapeutic and “theranostic” potential.

Khan et al. investigated the use of diosgenin, a natural compound, as an anti-inflammatory agent that also targets cancer and cancer-induced inflammation. They assessed the efficacy of Tween 80 (P80)-coated stearic acid solid lipid nanoparticles (SLNPs) encapsulating diosgenin in terms of stability, monodispersity, and entrapment efficiency, evaluating their potential as both anticancer and antidepressant agents. The study highlighted the use of diosgenin-incorporated SLNPs for enhancing grooming behavior and social interaction without any signs of toxicity. Further research is required to fully establish the safety profile of diosgenin and to evaluate the effectiveness of SLNPs in targeting specific conditions.

## Author contributions

NACL: Writing – original draft, Writing – review & editing. NS: Writing – review & editing. ST: Writing – review & editing.

